# Impact of Mediterranean Diet Adherence on Lipid Profiles in Pediatric Primary Dyslipidemia: Insights from the Updated KIDMED Score

**DOI:** 10.3390/nu17040623

**Published:** 2025-02-09

**Authors:** Giulia Massini, Nicolò Capra, Raffaele Buganza, Marta Vitello, Luisa de Sanctis, Ornella Guardamagna

**Affiliations:** 1 Department of Public Health and Pediatric Sciences, University of Turin, 10126 Turin, Italy; giulia.massini@unito.it (G.M.); raffale.buganza@unito.it (R.B.); luisa.desanctis@unito.it (L.d.S.); 2 Pediatric Endocrinology Unit, Regina Margherita Children’s Hospital, 10126 Turin, Italy; 3 Centro Cardiologico Monzino, IRCCS, 20138 Milan, Italy; nicolo.capra@cardiologicomonzino.it; 4 Department of Public Health and Pediatric Sciences, University of Medicine and Surgery of Turin, 10126 Turin, Italy; marta.vitello@edu.unito.it

**Keywords:** Mediterranean diet, pediatric dyslipidemia, KIDMED score, lipid profile, cardiovascular risk

## Abstract

**Background**: The Mediterranean diet (MD) has been shown to have cardioprotective effects, as demonstrated in adults, but data on hyperlipidemic children are scanty. This study assessed the impact of MD adherence, evaluated with the updated KIDMED score, on the lipid profiles of pediatric patients affected by primary hyperlipidemias. **Methods:** This retrospective study included data on 157 children (mean age: 10.01 ± 3.54 years) dating from 2016 to 2020. Dietary adherence and lipid levels were assessed at baseline (T_0_) and after 6 months (T_1_) of dietary counseling. Adherence was categorized using the KIDMED score: ≥8 (optimal), 4–7 (improvement needed), and ≤3 (very low). **Results:** KIDMED scores improved for 65% of patients, with adherence classes increasing for 33.8%. Significant reductions in LDL-C and non-HDL-C (*p* < 0.0001) levels were associated with even a one-point score increase, beyond which no additional benefits were observed. **Conclusions:** MD adherence, as measured using the updated KIDMED score, significantly improved the lipid profiles of children with dyslipidemia. These findings will support the performance of early dietary interventions to reduce cardiovascular risk factors.

## 1. Introduction

The prevention of atherosclerosis must begin at a pediatric age, as evidence strongly indicates that abnormal lipid levels during childhood are linked to the early development of atherosclerosis [1]. Furthermore, a clear relationship between the magnitude and duration of vascular exposure to low-density lipoprotein cholesterol (LDL-C) and the risk of cardiovascular (CV) disease (CVD) has been reported [2]. Diet is considered the cornerstone of lipid management for hyperlipidemic children, and nutritional interventions started in childhood also have a significant impact on eating habits in adulthood [3].

The Mediterranean diet (MD) has demonstrated effectiveness in improving lipid profiles and reducing CV events among adults [4,5], while pediatric studies on the MD have mostly focused on overweight and obesity [6], with limited data available on its effects in hypercholesterolemic children [7].

In 2004, Serra-Majem et al. [8] proposed the Mediterranean Diet Quality Index questionnaire (KIDMED score) to evaluate adherence to the MD among children and adolescents. This questionnaire is the most widely used scoring system for assessing MD adherence in the pediatric age group [9]. It consists of 16 points used to check the frequency of consuming different foods and explore eating habits. In 2019, Altavilla and Caballero-Perez [10] proposed an update of the KIDMED score, modifying some items to improve the estimation of compliance with the MD [Table 1]. This study did not find any correlations between lipid profiles and compliance class and the MD, but it had a restricted number of participants [11].

The aim of this study was to evaluate adherence to the MD according to the KIDMED score and its impacts on lipid profiles among children with primary hyperlipidemia, including familial hypercholesterolemia (FH) and polygenic dyslipidemia (PH).

## 2. Methods

### 2.1. Study Design and Participants

This study was a retrospective observational study conducted between 2016 and 2020. As per the enrollment criteria, we included hyperlipidemic pediatric patients affected by heterozygous familial hypercholesterolemia (HeFH) or poligenic hypercholesterolemia (PH) referred to the Outpatients Lipid Clinic of the Regina Margherita Children’s Hospital of Turin. Diagnoses were made following internationally recognized guidelines [1].

Family histories over two generations were reviewed to identify how the disorder was inherited. FH was diagnosed based on the following criteria: LDL-cholesterol levels ≥ 95th percentile (age, gender-specific), evidence of dominantly inherited high cholesterol, a family history of early cardiovascular events in a parent or grandparent, and the presence of an LDL receptor gene mutation. Children were classified as having PH if their LDL-C levels were above the 90th percentile but did not meet the criteria for inclusion in the FH group.

The exclusion criteria included secondary dyslipidemias, overweight or obesity (body mass index ≥ 85th percentile, adjusted for age and gender), kidney or liver disease, thyroid disorders (hyperthyroidism or hypothyroidism), high blood sugar or diabetes, immune–hematological disorders, or chronic illnesses requiring medication. Participants in the study were nonsmokers and had not been on any lipid-lowering treatments, including functional foods (e.g., phytosterols) or hypolipidemic drugs, prior to the study. Additionally, individuals without complete food diaries for both T_0_ and T_1_ were excluded. Families were also instructed not to change the children’s physical activity levels between T_0_ and T_1_.

This study involved 157 children (M/F 73/84) with a mean age of 10.01 ± 3.54 years, all diagnosed with primary dyslipidemia. At the time of diagnosis, and for at least six months prior to the study, the children had already transitioned from a free diet to the CHILD I nutritional protocol. During the study, participants attended two visits: one at baseline (T_0_) and another six months later (T_1_). These visits included clinical and auxological evaluations, serum lipid level analysis, and a review of dietary records. At T_0_, all participants were instructed to adhere to a Mediterranean diet (MD). Dietary habits were assessed through self-reported weekly food diaries, and the KIDMED questionnaire was administered at both T_0_ and T_1_.

Serum lipids were analyzed from blood samples taken after a 12 h fasting period using an automated method (Integra Plus 400/Cobas 6000) with commercial kits (Automated Modular P3 Analyzer, Roche(Roche Diagnostics S-p-A., Monza, Italy). LDL-C levels were calculated using the Friedewald equation, namely, TC − HDL-C − (TG/5) [12], while non-HDL-C was determined by subtracting HDL-C from TC.

This study was conducted in compliance with the Declaration of Helsinki and received approval from the Local Ethics Committee. Written informed consent was obtained from all participants and their legal guardians.

### 2.2. Mediterranean Diet and KIDMED Score Application

The Mediterranean diet (MD) reflects the traditional dietary habits of Euro-Mediterranean countries, incorporating variations influenced by geography, culture, and local food products. The core principles of the MD focus not only on specific types of foods but also on balanced daily nutrient intake and lifestyle factors, emphasizing a holistic approach to eating. The recommended macronutrient distribution aligns with recognized guidelines: one should acquire 55–60% of their total energy from carbohydrates (with 80% being complex carbohydrates), 10–15% from proteins (approximately 60% of which is of animal origin), and 25–30% from fats, predominantly sourced from olive oil [13,14,15].

The MD is centered around eating healthy, whole foods, with a focus on cereals, fruits, and vegetables. Meals typically include 1–2 servings of cereals, especially whole grains like whole-grain bread, pasta, rice, oats, barley, and spelt, which are rich in fiber. Fruits (1–2 servings per meal) and vegetables (at least 2 servings per meal) are also key, providing vitamins, minerals, and antioxidants. Healthy fats in the MD mainly come from olive oil (1–2 servings per meal), along with olives, seeds, and nuts (1–2 servings per day). Protein sources include fish (at least 2 servings per week), white meat (2 servings per week), and eggs (2–4 servings per week). Legumes are a great plant-based protein, with a recommended intake of at least 2 servings per week. For dairy, it is best to consume low-fat options like yogurt and other fermented dairy products in moderation (2 servings per day), as they provide calcium and nutrients that are beneficial for bone and heart health. The diet also suggests limiting consumption of red meat (less than 2 servings per week), processed meats (no more than 1 serving per week), and junk food, snacks, and sweets (no more than 2 servings per week) in order to reduce intake of unhealthy fats, salt, sugars, and additives [13,14].

In this study, participants and their families were provided with a weekly food diary form and asked to complete it during the week leading up to visits at both T_0_ and T_1_, when the form was handed out. The diary had three sections, corresponding to the main meals of the day: breakfast, lunch, and dinner. For each meal, participants were required to specify the types of food consumed and the corresponding quantities, either by weight or using common measures such as tablespoons, teaspoons, cups, or bowls. They were also asked to note any other foods not listed, particularly morning and afternoon snacks. During the first visit, participants and their parents received dietary counselling according to the Mediterranean diet style from an expert nutritionist in a face-to-face meeting. A second nutritional assessment was performed 6 months after the first evaluation. During this visit, the diary report was discussed with the nutritionist to verify the quality and accuracy of compliance with the dietary indications of the Mediterranean diet to ascertain any shortcomings or doubts that would then have been evaluated in detail. Subsequently, the Mediterranean Diet Quality Index was applied to evaluate MD adherence at T_0_ and T_1_.

On the basis of their final scores, patients were assigned to one of three adherence classes: ≥8, indicating optimal Mediterranean diet adherence; 4–7, indicating improvement is needed to adjust one’s intake to Mediterranean patterns; and ≤3, indicting very low diet quality [8,10]. In the statistical analysis, for the change in the adherence score (∆), we considered three stages: ∆ KIDMED Score = 0; ∆ KIDMED Score +1; and ∆ KIDMED Score ≥ +2.

**Figure 1:**
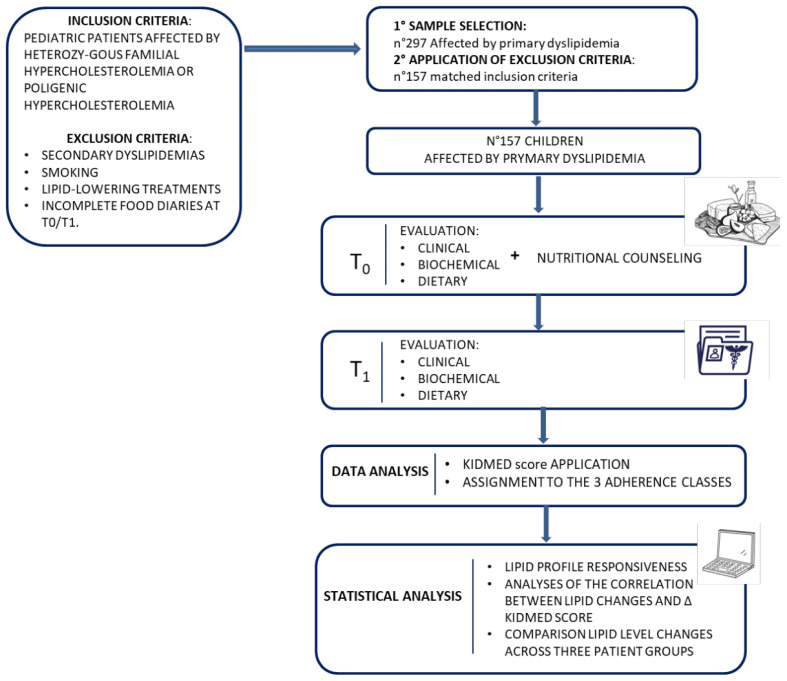
Study flowchart.

A flowchart for this study is shown in Figure 1.

### 2.3. Statistical Analysis

Continuous variables are expressed as means ± standard deviations (SD), skewed variables are given as medians (25th–75th percentile), and categorical variables are shown as absolute numbers and percentages.

A paired *t*-test or a Wilcoxon signed-rank test was applied to evaluate differences for these variables between T_0_ and T_1_.

Multivariate linear regression model was employed to adjust the relationship between each percentage change in lipid levels (∆% non-HDL-c, ∆% LDL-c and ∆% TC) and variation in KIDMED scores (∆ KIDMED Score) for some confounders (age, gender, time between the two measurements, and type of diagnosis). After checking the homogeneity of variance using Levene’s test, a General Linear Model (GLM) was employed to analyze the differences in the percentage change in lipid levels (non-HDL, LDL-c, and TC) among three groups of patients: those who increased their adherence score by 2 points, those who increased it by 1 point, and those who did not improve their adherence score. Bonferroni’s correction for multiple comparisons between groups was applied. All tests were 2-tailed, and *p* < 0.05 was considered statistically significant. All analyses were performed using SAS version 9.4 (SAS Institute, Cary, NC, USA).

## 3. Results

The main characteristics of the survey population at baseline and at the end of the study are summarized in Table 2. All 157 participants enrolled participants successfully completed this study.

At baseline, the KIDMED scores were optimal, medium, and low for n. 1 (0.6%), n. 76 (48.4%), and n. 80 (51%) patients, respectively, while at the end of the study, that is, six months later, the KIDMED score improved for n. 102 patients (65%), was unchanged for n. 14 (18.5%), and worsened for n. 26 (16.6%). Among the patients who improved their scores, n. 52 (51%) of them had scores that increased by 1 point, and n. 28 (27%) patients’ scores increased by 2 points. Among those whose scores worsened, n. 18 (69%) of them had scores that dropped by 1 point, and n. 13 (19%) had scores that dropped by 2 points (Table 3).

In Table 4, the variation in adherence classes following nutritional counseling can be observed. Specifically, 29 children moved from the low-adherence and higher-adherence classes (n. +18 and n. +11, respectively) to the medium- and optimal-adherence classes.

Table 5 shows changes in the adherence classes following the dietary intervention.

Improvements in KIDMED scores were significantly associated with reductions in non-HDL, LDL-C, and TC levels (*p* < 0.0001 adjusted for age, gender, time between the two measurements, and diagnosis). (Table 6 (a,b)). In contrast, no significant associations between HDL-c and KIDMED score were observed (*p*: 0.068).

No statistically significant difference was observed in the percentage variation of non-HDL and LDL-C levels between patients with a 1-point increase and those with a ≥ +2-point increase in their KIDMED scores. However, both 1-point and ≥+2-point increases were significantly associated with a greater reduction in non-HDL, LDL-C, and TC levels compared to the levels for patients with no improvement in KIDMED scores (Table 7).

## 4. Discussion

The present paper focuses on the application of the KIDMED score to evaluate the closeness with which children with primary hyperlipidemia adhere to the MD. Assessing nutrient intake is a complex task, and food scores are widely used for adults as an alternative to direct qualitative measurements.

The KIDMED score is a straightforward and practical tool used to assess children’s dietary habits. The updated version of the index used in this study includes some modifications from the original. For example, Item 1 now asks if a child “eats fruit every day”, replacing the previous question about drinking fruit juice daily. Item 8 has been changed to inquire if a child “eats whole-grain pasta or rice almost every day”, instead of the original item about consuming pasta or rice. Additionally, Item 9 now focuses on whether a child “eats whole cereals or whole grains for breakfast”, replacing the previous item about eating cereal or cereal products. These changes are designed to reduce the intake of simple sugars and promote the consumption of whole grains, which are considered healthier [10].

The benefits of the MD have been extensively documented. In 1965, Ancel Keys first reported a reduction in cardiovascular risk in populations whose nutritional habits aligned with the MD [16]. Subsequent studies have confirmed the benefits of the MD for adults, including reductions in cardiovascular and cerebrovascular diseases, cancer, improved glycemic control, and enhanced cognitive function [14,17,18,19,20].

A decline in adherence to the Mediterranean diet (MD) may contribute to an increase in chronic non-communicable diseases starting in childhood. Iaccarino et al. conducted a systematic review aimed at synthesizing the existing literature on MD compliance among children and adolescents [9]. They highlighted that MD adherence varied significantly among children and adolescents across Mediterranean countries and showed notable differences across European countries, with limited data available for non-Mediterranean regions. The lowest prevalence of subjects with low MD adherence (<10%) and the highest prevalence of those with high adherence were observed in some Spanish studies. In particular, in 30 studies, the distribution of KIDMED categories (poor, average, and good adherence) was analyzed: poor adherence ranged from 1.6% among Spanish children to 62.8% among Greek adolescents, average adherence varied from 28.0% among Greek adolescents to 73.8% among rural Italian adolescents, and good adherence ranged from 4.3% among Greek children aged 10–12 to 53.9% among Spanish children [9].

However, studies on the MD in relation to children are limited, primarily focusing on overweight populations [21,22,23]. In 2018, Archero et al. conducted a study evaluating differences in adherence to the MD among primary and secondary school children and adolescents in Northern Italy as well as its associations with weight status [21]. The cited study assessed adherence using the KIDMED questionnaire among n. 669 participants aged 6 to 16 years. The findings revealed that adherence was low (scores ≤ 3) among 16.7%, moderate (scores 4–7) among 63.7%, and high (scores ≥ 8) among 19.6% of the students. Poor-quality food is replacing food in the Mediterranean diet, particularly among younger children, leading to an increased risk of overweight [21]. A recent study by Warkentin et al. sought to identify dietary patterns in childhood and investigate their relationship with adiposity-related outcomes during childhood and adolescence [22]. It included data from six European countries (Spain, France, UK, Greece, Lithuania, and Norway). Closeness to the MD was calculated through KIDMED scores during childhood (*n* = 1597) and adolescence (*n* = 803). Norwegian children demonstrated better overall diet quality; in contrast, Lithuanian children exhibited the poorest diet quality, with higher consumption of sweets and the highest adherence to the “Western pattern”. Lower intake of healthy foods was associated with higher adiposity in children, with reduced “Healthy pattern” adherence being linked to increased fat mass [22].

Few studies have been conducted on the pediatric population regarding the relationship between the Mediterranean diet and cardiovascular risk; however, some studies have estimated the MD’s anti-inflammatory effects. Kanısoy et al. conducted an interesting cross-sectional study in Northern Cyprus between 2019 and 2020, involving 300 primary school children, with the aim of investigating the relationship between Diet Inflammatory Index (DII) scores and dietary quality, assessed using the KIDMED score. Their study highlighted the anti-inflammatory properties of the MD, showing clear positive effects on diet quality and anthropometric measurements. Moreover, they suggested that the adherence to MD reduces the risk of chronic diseases through improving DII scores from an early age [24]. In 2021, Augimeri et al. used the KIDMED score to assess MD adherence, nutrient intake, and serum antioxidant/anti-inflammatory properties among adolescents [25]. Participants were grouped by MD adherence, revealing higher PUFA/SFA ratios, intakes of dietary fibers and vitamins, and oxygen radical absorbance capacity in the optimal adherence group. Serum C-reactive protein levels were negatively correlated with PUFA/SFA ratios, while total ORAC was positively correlated with glucose. Reactive oxygen metabolite concentrations were lower in the optimal-adherence groups. Serum from adolescents who strongly adhered to the MD showed reduced pro-inflammatory cytokine secretion, highlighting its potential to prevent chronic diseases [25]. A recent study conducted in 2024 by Cordos et al. used another tool to assess inflammatory status and oxidative stress in hemodialysis patients to detect early signs of inflammation and malnutrition: the Prognostic Inflammatory and Nutritional Index (PINI). The Prognostic Inflammatory and Nutritional Index (PINI) was calculated using the following formula: alpha1-acid glycoprotein (AGP) × C-reactive protein (CRP)/albumin (ALB) × transthyretin (TTR). A PINI score of less than 1 was classified as normal. This study demonstrated how the PINI is an effective tool for detecting occult malnutrition–inflammation syndrome and predicting the risk of acute events [26].

Our previous research demonstrated the efficacy of the MD in reducing LDL-C levels in hyperlipidemic children [27]. The positive effects of the MD have been attributed to several factors, including fiber and antioxidants from fruits, vegetables, and whole grains; a high intake of unsaturated fatty acids from olive oil, nuts, olives, and fish; and reduced consumption of animal-derived fats and cholesterol through lean meats and low-fat dairy products [6,7,27]. Given the emphasis on food quality, the KIDMED score is an appropriate method of measuring the closeness of adherence to the MD. In contrast, quantitative measurements of macronutrients provide detailed data on carbohydrates, proteins, and fats without considering the food from which they are sourced, thus failing to account for overall dietary patterns. Additionally, quantitative approaches require detailed food diaries spanning 3–7 days, making them time-intensive and burdensome for families and healthcare professionals.

In this study, most of the participants demonstrated compliance, leading to significant improvements in MD adherence compared to the baseline, as shown by the total KIDMED score. This improvement was related to reductions in atherogenic lipoprotein levels. Notably, adherence class changes revealed important insights: the proportion of participants in the low-adherence category decreased from 51% to 32.5%. It is interesting to note that out of n. 80 (51%) patients with very low diet quality at baseline, n. 43 (53.8%) improved their classes, while n. 37 (46.2%) remained in the low one, 52.5% moved to medium adherence, and 1.25% transitioned to the optimal adherence category. Furthermore, of the 76 patients falling under the medium adherence class at T_0_, n. 10 (13.2%) ascended to the optimal one, n. 52 (68.4%) maintained their adherence class, and n. 14 (18.4%) dropped into the low one (Table 5). This shift is significant, as the increase in adherence score (∆ adherence) was strongly associated with reductions in LDL-C and non-HDL-C levels in multivariate analysis, serving as a predictive factor for lipid profile improvement.

A key question concerns the quantitative score variation needed to achieve lipid profile changes. Our findings indicate that an increase of at least one adherence-score point positively impacts total cholesterol, LDL-C, and non-HDL-C levels, although HDL-C remained unaffected. These results underscore the utility of the KIDMED score in monitoring dietary adherence and the effectiveness of the MD in facilitating metabolic improvements. Reducing LDL-C and non-HDL-C levels is particularly critical in managing inherited primary hyperlipidemia.

This study uniquely evaluates MD adherence and its impact on lipid profiles among children with cardiovascular risk as affected by primary dyslipidemias, constituting a less-explored area when compared to adults. Using an updated KIDMED score, we have shown that even a one-point improvement significantly reduces LDL-C and non-HDL-C levels, highlighting a dose-dependent relationship. The findings emphasize the value of early cardiovascular prevention and support the use of dietary counseling as a non-invasive, accessible strategy to manage pediatric dyslipidemia, reducing reliance on pharmacological treatments.

However, we must highlight some limitations: this study’s retrospective design limits the ability to make causal inferences, and the 6-month follow-up may not have captured long-term adherence or lipid improvements; furthermore, the KIDMED score, while validated, relies on self-reported data prone to recall bias, especially with respect to younger children. Future research should include randomized controlled trials (RCTs) to confirm causal relationships and allow comparison of the MD with other diets. Additionally, longer follow-up periods should be used to assess sustainability and long-term impacts on cardiovascular outcomes.

## 5. Conclusions

This study demonstrates the effectiveness of the updated KIDMED score as a practical tool for assessing adherence to the Mediterranean diet (MD) among children with primary hyperlipidemia. Improved MD adherence was linked to significant reductions in LDL-C and non-HDL-C levels, key targets in hyperlipidemia management. The findings reaffirm the MD’s benefits as a nutrient-rich, heart-healthy dietary approach. The KIDMED score offers a simple, reliable alternative to complex quantitative dietary assessments, emphasizing its value in clinical and public health settings.

## Figures and Tables

**Table 1:** KIDMED—Mediterranean Diet Quality Index for children and adolescents (updated version) [8].

KIDMED Index	Score
1. Eats a serving of fruit every day	+1
2. Has a second serving of fruit every day	+1
3. Eats fresh or cooked vegetables regularly once a day	+1
4. Eats fresh or cooked vegetables more than once a day	+1
5. Consumes fish regularly (at least 2–3 times per week)	+1
6. Goes more than once a week to a fast-food (e.g., hamburger) restaurant	−1
7. Likes pulses and eats them more than once a week	+1
8. Consumes whole-grain pasta or whole-grain rice almost every day (5 or more times per week)	+1
9. Consumes whole cereals or whole grains (whole-meal bread, etc.) for breakfast	+1
10. Consumes nuts regularly (at least 2–3 times per week)	+1
11. Uses olive oil at home	+1
12. Skips breakfast	−1
13. Has a dairy product for breakfast (yogurt, milk, etc.)	+1
14. Has commercially baked goods or pastries for breakfast	−1
15. Eats two yogurt servings and/or some cheese (40 g) daily	+1
16. Eats sweets and candy several times every day	−1

**Table 2:** Participants’ characteristics at baseline and at the end of study.

Variable	T_0_	T_1_	*p*-Value
Gender (Male)	73 (46.5%)	–	–
Age (Years)	10.01 ± 3.54	–	–
BMI (Kg/m^2^)	19.01 ± 3.8	19.15 ± 3.73	0.1003
TC (mg/dL)	216 (195; 253)	204 (184; 245)	<0.0001
Tg (mg/dL)	68 (55; 99)	74 (54; 99)	0.692
HDL-c (mg/dL)	58.1 ± 13.87	56.90 ± 13.43	0.1474
LDL-c (mg/dL)	137 (119; 181)	128 (110; 177)	<0.0001
Non-HDL-c (mg/dL)	157 (136; 193)	147 (128; 191)	<0.0001
KIDMED SCORE	3.61 ± 1.47	4.56 ± 1.83	<0.0001

Categorical variables are reported as absolute numbers (percentages), while numerical variables are reported as means ± standard deviations or medians (25th and 75th percentiles).

**Table 3:** The ∆ KIDMED scores after dietary counseling.

Score Variation	∆ KIDMED Score	Frequency (n°)	TOT (n°)	%
Worse	−4	2	n.26	16.6%
	−3	1		
	−2	5		
	−1	18		
Unchanged	0	29	n.29	18.5%
Improved	+1	52	n.102	65.0%
	+2	28		
	+3	13		
	+4	4		
	+5	5		

**Table 4:** Adherence classes of participants (tot. n. 157, %) and class changes at baseline and at the end of study.

Variable	Low n. (%)	Medium n. (%)	Optimal n. (%)
T_0_	80 (51)	76 (48.4)	1 (0.6)
T_1_	51 (32.5)	94 (59.9)	12 (7.6)
∆T_0_ − T_1_ (% of the total)	−29 (18.47)	+18 (11.46)	+11 (7)

**Table 5:** Adherence class variation after dietary counseling.

		Adherence Class T_1_			
		Low n.	Medium n.	Optimal n.	Total n.
Adherence class T_0_	Low n.	**37**	**42**	**1**	80
	Medium n.	**14**	**52**	**10**	76
	Optimal n.	**0**	**0**	**1**	1
	Total n.	51	94	12	157

Green boxes refer to participants who improved their adherence classes; pink boxes refer to participants whose adherence classes remained unchanged; red boxes refer to participants whose adherence classes worsened. n.: number of patients.

**Table 6:** Adjusted association between changes in KIDMED scores and percentage change in (a) non-HDL-C, (b) LDL-C and (c) TC levels.

**(a) Multivariate Linear Model for Percentage Change in Non-HDL-C Levels (∆% Non-HDL-C)**				
	**β**	**95% CL**		***p*-Value**
Time between measurements	0.235	−2.153	2.622	0.8461
Age T_0_	−0.075	−0.608	0.458	0.7818
Gender (Male)	−2.415	−6.130	1.301	0.2011
Type of diagnosis (PH)	0.549	−3.299	4.397	0.7783
∆ KIDMED score	−3.882	−5.070	−2.695	<0.0001
**(b) Multivariate Linear Model for Percentage Change in LDL-C Levels (∆% LDL-C)**				
	**β**	**95% CL**		***p*-Value**
Time between measurements	0.196	−2.468	2.860	0.8845
Age T_0_	−0.204	−0.799	0.391	0.4988
Gender (Male)	−0.889	−5.034	3.257	0.6725
Type of diagnosis (PH)	−0.161	−4.454	4.132	0.941
∆ KIDMED score	−4.585	−5.909	−3.260	<0.0001
**(c) Multivariate Linear Model for Percentage Change in TC (** **∆% TC)**				
	**β**	**95% CL**		***p*** **-Value**
Time between measurements	1.047	−1.014	3.109	0.317
Age T_0_	0.122	−0.339	0.582	0.6026
Gender (Male)	−2.134	−5.341	1.074	0.1908
Type of diagnosis (PH)	0.032	−3.291	3.354	0.9851
∆ KIDMED score	−3.279	−4.304	−2.254	<0.0001

The regression coefficient (β) represents the estimated percentage change in (a) non-HDL-C, (b) LDL-C and (c) TC levels for a one-unit increase in continuous variables (i.e., time between measurements, age, and ∆ KIDMED score), with all other variables remaining constant. For categorical variables, β indicates the difference in percentage change relative to the reference: males vs. females (gender) and PH vs. FH (type of diagnosis).

**Table 7:** Comparison of mean percentage change in non-HDL, LDL-C, and TC levels between ∆ KIDMED score groups.

Variable	∆ KIDMED Score ≥ +2	∆ KIDMED Score = +1	∆ KIDMED Score ≤ 0	*p*-Value ^a^	*p*-Value ^b^	*p*-Value ^c^
∆% non-HDL-C	−10.4% (−13.8%; −7%)	−7.8% (−11.1%; −4.4%)	2.3% (−1.1%; 5.7%)	0.828	<0.0001	0.0002
∆% LDL-C	−11.8% (−15.6%; −8%)	−9.2% (−13%; −5.5%)	3.3% (−0.5%; 7.1%)	1	<0.0001	<0.0001
∆% TC	−8.8% (−11.7%; −5.8%)	−6.7% (−9.6%; −3.8%)	2.3% (−0.7%; 5.2%)	0.947	<0.0001	<0.0001

Adjusted means (95% CL) for age, gender, time between the two measurements, and type of diagnosis. *p*-value ^a^: comparison between ∆ KIDMED score ≥ +2 and ∆ KIDMED score = +1; *p*-value ^b^: comparison between ∆ KIDMED score ≥ +2 and ∆ KIDMED score ≤ 0; *p*-value ^c^: comparison between ∆ KIDMED score = +1 and ∆ KIDMED score ≤ 0.

## Data Availability

The records of patients are available in our local Informatic Database Trak-Care System.
